# From “one medicine” to “one health” and systemic approaches to health and well-being^☆^

**DOI:** 10.1016/j.prevetmed.2010.07.003

**Published:** 2011-09-01

**Authors:** J. Zinsstag, E. Schelling, D. Waltner-Toews, M. Tanner

**Affiliations:** a Swiss Tropical and Public Health Institute/University of Basel, PO Box, CH-4002 Basel, Switzerland; b Ontario Veterinary College, University of Guelph, 50 Stone Road, Guelph, ON, Canada N1G 2W1

**Keywords:** Human health, Human medicine, Animal health, Veterinary medicine, Cooperation, “One medicine”, “One health”, Veterinary public health, “Ecosystem health”, Systems biology, Social-ecological systems

## Abstract

Faced with complex patterns of global change, the inextricable interconnection of humans, pet animals, livestock and wildlife and their social and ecological environment is evident and requires integrated approaches to human and animal health and their respective social and environmental contexts. The history of integrative thinking of human and animal health is briefly reviewed from early historical times, to the foundation of universities in Europe, up to the beginning of comparative medicine at the end of the 19th century. In the 20th century, Calvin Schwabe coined the concept of “one medicine”. It recognises that there is no difference of paradigm between human and veterinary medicine and both disciplines can contribute to the development of each other. Considering a broader approach to health and well-being of societies, the original concept of “one medicine” was extended to “one health” through practical implementations and careful validations in different settings. Given the global health thinking in recent decades, ecosystem approaches to health have emerged. Based on complex ecological thinking that goes beyond humans and animals, these approaches consider inextricable linkages between ecosystems and health, known as “ecosystem health”. Despite these integrative conceptual and methodological developments, large portions of human and animal health thinking and actions still remain in separate disciplinary silos. Evidence for added value of a coherent application of “one health” compared to separated sectorial thinking is, however, now growing. Integrative thinking is increasingly being considered in academic curricula, clinical practice, ministries of health and livestock/agriculture and international organizations. Challenges remain, focusing around key questions such as how does “one health” evolve and what are the elements of a modern theory of health? The close interdependence of humans and animals in their social and ecological context relates to the concept of “human-environmental systems”, also called “social-ecological systems”. The theory and practice of understanding and managing human activities in the context of social-ecological systems has been well-developed by members of The Resilience Alliance and was used extensively in the Millennium Ecosystem Assessment, including its work on human well-being outcomes. This in turn entails systems theory applied to human and animal health. Examples of successful systems approaches to public health show unexpected results. Analogous to “systems biology” which focuses mostly on the interplay of proteins and molecules at a sub-cellular level, a systemic approach to health in social-ecological systems (HSES) is an inter- and trans-disciplinary study of complex interactions in all health-related fields. HSES moves beyond “one health” and “eco-health”, expecting to identify emerging properties and determinants of health that may arise from a systemic view ranging across scales from molecules to the ecological and socio-cultural context, as well from the comparison with different disease endemicities and health systems structures.

## 1 Introduction

In the past decade health research for humans and animals has been confronted with increasingly complex issues of global change which may supersede primary health concerns in the magnitude of their leverage. Most of these issues are concomitant with the increase in human population and its ramifications of rapid urbanisation, intensified livestock production, encroachment of ecosystems and globalised trade and traffic. Moreover, in the last few years unprecedented increases in food and energy prices have threatened to jeopardize fragile improvements in the economies of many developing countries (www.fao.org/news/story/en/item/28797/icode/ accessed 01.30.10). Looming crises with regards to the use of natural resources and raw materials, and in particular access to and use of water, threaten to become sources of conflict and may lead to open warfare (World Water Assessment Programme; www.unesco.org/water/wwap/ accessed 07.22.10). In contrast to the objectives of the Millennium Development Goals, the socio-economic divide between developing and industrialized countries is growing (www.un.org/milleniumgoals/ accessed 07.22.10). In several areas of the world, state governance has failed or even collapsed, leading to rapidly increasing labour migration across continents, claiming the lives of hundreds of unfortunate migrants, e.g. in the Mediterranean. In addition, the effects of climate change become visible and challenge the coping strategies of the most vulnerable coastal populations (www.ipcc.ch/publications_and_data/ar4/wg2/en/contents.html accessed 07.22.10 Parry et al., 2007).

Faced with these complex, often rapidly changing patterns, the inextricable interconnection of humans, pet animals, livestock and wildlife and their social and ecological environment is evident and requires integrated approaches to human and animal health and their respective social and environmental contexts. This paper firstly recalls briefly the history of integrative thinking on human and animal health, secondly it reviews “one medicine” and “ecosystem approaches to health” in the conceptual landscape of comparable and neighbouring approaches, and thirdly it explores avenues of systemic approaches to the health of animal and humans and their potential to address the challenges ahead.

## 2 Brief history of integrative thinking on human and animal health

Ancient healers were often priests and cared for both humans and animals (Schwabe, 1984). They gained anatomical and pathological skills from slaughtering sacrificial animals and deciding on their purity for sacrifice (Leviticus 1,3). Egyptian papyri deal with human and animal diseases (Papyrus of Kahun, ca. 1800 BC) (Bardinet, 1995; Driesch and Peters, 2003), seeing humans and animals as the “flock of God” and having chimeric human and animal creatures in their mythology. Medical knowledge in India is influenced by beliefs about metempsychosis (transmigration) and reincarnation between animals and humans. Veterinary medicine appears to have been a distinct discipline during the Zhou Dynasty in China (11–13th century). The Zhou Dynasty had one of the earliest organizations of an integrated public health system including medical doctors and veterinarians (Driesch and Peters, 2003). A Chinese text by Xu Dachun (‘On the origin and development of medicine’) from the 18th century states that: “The foundations of veterinary medicine are as comprehensive and subtle as those of human medicine and it is not possible to place one above the other” (translated from German (Driesch and Peters, 2003)). In the sphere of Arab influence, medical science reached a culminating point towards the end of the first millennium with specific hippiatric texts like the Kitab al Baytara (). Human medicine was integrated into the medieval universities, whereas veterinary medicine remained largely in the hands of equerries until the 18th century (Rüegg, 2004). Claude Bourgelat, the founder of the first veterinary school in Lyon in 1762, was heavily criticised when he recommended human clinical training for the veterinary curriculum (Driesch and Peters, 2003). However, in the 19th century, with the advent of cellular pathology, scientists like Rudolf Virchow developed a strong interest in linking human and veterinary medicine as a form of comparative medicine based on the discovery of similar disease processes in humans and animals (Saunders, 2000). For example, major animal diseases such as rinderpest, rather than human epidemics, were the stimulus for medical research in South Africa and tsetse fly (*Glossina* spp.) control was motivated primarily by cattle trypanosomiasis (Dukes, 2000).

## 3 “One medicine”

**Fig. 1:**
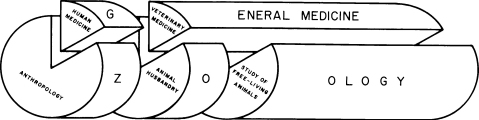
Calvin Schwabe's “one medicine” as general medicine of humans, domestic and free-living animals (reproduced with permission from Schwabe, 1984).

Integrated medical thinking was conveyed to North America by William Osler, a student of Virchow. He is credited for having coined the term “one medicine” (Dukes, 2000), although no direct written evidence for this has been found (Cardiff, R.D., personal communication). In the 20th century, both sciences specialised to such an extent that their association was hardly visible and less often practised. It was Calvin Schwabe's thorough rethinking of the concept of “one medicine” in 1976 that fully recognised the close systemic interaction of humans and animals for nutrition, livelihood and health (Schwabe, 1984). Today, the earliest forms of healing of humans and animals are still widely practised in traditional pastoral societies. It is thus not surprising that the contemporary “one medicine” idea grew out of experiences in African communities. It was conceived and conceptually consolidated during Calvin Schwabe's work with Dinka pastoralists (Majok and Schwabe, 1996) but also represents myths of co-creation of humans and cattle from Fulbe pastoralists in West Africa (Anonymous, 1966). It basically means that there is no difference of paradigm between human and veterinary medicine, and is an extension of notions of comparative medicine that were prevalent in North American veterinary and medical schools in the 1970s and 1980s. Both sciences share, as a general medicine, a common body of knowledge in anatomy, physiology, pathology, and the origins of diseases in all species (Fig. 1) (Schwabe, 1984). For example, close genomic relationship of animals and humans exists in cancer genetics, and many cancer genes were discovered in animals prior to identifying similar pathologies in humans. Currently, functional genomics of human and animal genes are pulled together by a huge International Knockout Mouse Consortium (IKMC, www.knockoutmouse.org). Such cross-over work should, however, not lead to an “Other one medicine”, but should contribute to the convergence of an integrated approach to health of all species (Cardiff et al., 2008).

## 4 “One health”

International organizations such as the World Health Organization (WHO) and the Food and Agriculture Organization (FAO) have institutionalised “one medicine” partly as Veterinary Public Health (VPH), the contribution of veterinary medicine to public health. The concept of “ecosystem health” extends “one medicine” to the whole ecosystem, including wildlife. Sustainable development depends on the mutualism of health and well-being of humans, animals and the ecosystems in which they co-exist (Rapport et al., 1998, 1999; Forget and Lebel, 2001; Lebel, 2002). Conservationists have recognised and promoted what are known as the “Manhattan principles” (www.oneworldonehealth.org), that the health and sustainable maintenance of wildlife in natural reserves are mutually interdependent with the health of communities and the livestock surrounding them (Osofsky et al., 2005). Finally, many of the causative agents with bio-terrorism potential are zoonoses and hence require mutual animal and public health vigilance for rapid detection (Kahn, 2006). The term “one medicine”, having a rather clinical connotation, reflects insufficiently the interactions between human and animal health that reach far beyond individual clinical issues and include ecology, public health and broader societal dimensions (Zinsstag et al., 2005b). “One medicine” evolves thus towards “one health” through practical implementation and careful validation of contemporary thinking on health and ecosystems and their relevance for global public and animal health development (Zinsstag et al., 2005b, 2009b; Zinsstag and Tanner, 2008). A strategic framework for reducing risks of infectious diseases at the animal-human-ecosystem interfaces was first released at the 6th International Ministerial Conference on Avian and Pandemic Influenza in Sharm el-Sheikh, in October 2008 (FAO et al., 2008) and has further evolved under the trademark protected term “One World One Health™” during an expert consultation in Winnipeg, Canada in 2009 (Anonymous, 2009).

## 5 “One health” road work ahead

“One health” has seen unprecedented revival in the last decade with fostered awareness, scientific debate, research programmes (www.onehealthcommission.org), integrated disease surveillance (www.promedmail.org) and an open toolbox in the fields of disease surveillance, epidemiological studies and health care provision (Zinsstag et al., 2009b). Despite all efforts of cooperation between human and animal health, isolated silo thinking persists, particularly in the public health sector. For example, in the disputed case of human to pig transmission of H1N1 in Alberta, Canada in early 2009, an official of the Canadian Food Safety Agency (CFIA) complained about the lack of cooperation with human health counterparts in testing involved people (Branswell, 2009). How can the public health sector perceive advantages of using “one health”? Demonstrating evidence of an added value of “one health” compared to conventional separated sectoral approaches is the major task that lies ahead, and represents the unfinished agenda of “one health” in view of further systemic conceptual developments (see below). Further evidence of public health benefits by interventions in animals (Roth et al., 2003; Zinsstag et al., 2009a), by joint health care provision (Schelling et al., 2005), or by joint disease surveillance (www.who.int/mediacentre/news/new/2006/nw02/en/index.html), should be generated to foster interactions between human and animal health at the academic level (www.onehealthinitiative.com), in ministries (www.phax-aspc.gc.ca/cipars-picra/index-eng.php) and in international organizations in industrial and developing countries (Zinsstag and Tanner, 2008). In our view, “one health” can hardly be claimed as a trademark protected term, because it is only one element in the above mentioned conceptual thinking, which is much broader and goes beyond the direct animal–human interconnectedness, including eco-health, agro-ecosystem health, resilience, adaptive management and sustainability studies. A stream of multidisciplinary research for sustainable development uses the term “syndromes of global change”, which includes health of animals and humans as a systems component (www.nccr-north-south.unibe.ch).

## 6 Ecosystem approaches to health

“Ecosystem approaches to health” or “eco-health” considers inextricable linkages between ecosystems, society and health of animals and humans (Rapport et al., 1998, 1999). In-depth understanding of ecological processes allowed, for example, to show that mercury poisoning of fish and impeding health risks for humans in the Amazon basin were not due to upstream gold mining but due to soil erosion following deforestation (Forget and Lebel, 2001). Similarly, changes in patterns of malaria in Central America and the emergence of *Cyclospora* infections in North America have been related to United States foreign policy initiatives in the 1980s (Waltner-Toews, 1999). Such examples demonstrate that contemporary complex health problems cannot be solved by reductionist approaches and that they require systems thinking, which are promoted by the International Association of Ecology and Health (www.ecohealth.net).

Despite these integrated approaches to health, we observe an ongoing and accelerated fragmentation of veterinary and medical science into a large number of sub-disciplines with an increasing risk of misinterpretation in diagnosis and pathology (Cardiff et al., 2008). There is an exponentially increasing mass of information that cannot be overseen by individuals. At the same time we recognise the complex interdependence of humans, animals and their environment. Attempts at global overviews threaten to become meaningless, and mainstream “reductionist” research can only explain phenomena at a very small scale. How can we move towards a modern theory of health of animals and humans which has the power to deal with new complex challenges of global change ahead?

## 7 Towards a systems approach to health of animals and humans

**Fig. 2:**
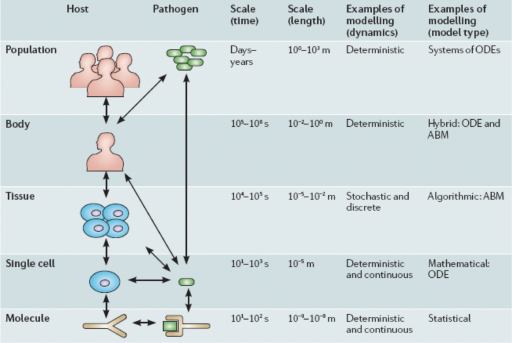
Scales of *Mycobacterium tuberculosis* infection and their system interactions. Reprinted by permission from Macmillan Publishers Ltd: Nature Microbiology Reviews, Young et al. (2008), copyright (2008).

**Fig. 3:**
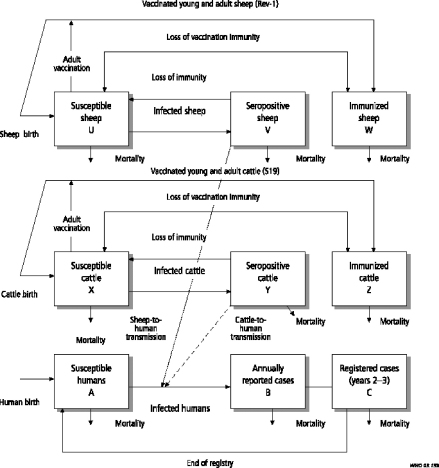
Livestock to human transmission of brucellosis (reproduced with permission from Roth et al., 2003).

**Fig. 4:**
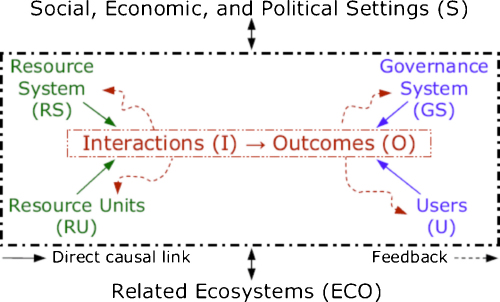
A multitier framework for analyzing Socio-Ecological Systems SES (Ostrom, 2007, Copyright (2007) National Academy of Sciences, U.S.A.).

**Fig. 5:**
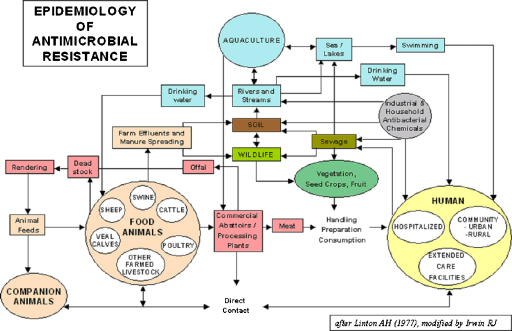
Flow chart of antimicrobial resistance of the Canadian Integrated Programme for Antimicrobial Resistance Surveillance (CIPARS) www.phac-aspc.gc.ca/cipars-picra/index-eng.php. Irwin (2005) adapted from Linton (1977) (personal communication by Rebecca Irwin 07.21.2010).

Systems thinking involves non-linear relationships and feed-back loops between components of complex entities at different scales. Recent work in the fields of systems biology, zoonosis control, public health, ecology and the social sciences demonstrates quantitative and qualitative systemic linkages ranging from populations to molecular processes. Although much systems biology has focused on the cellular and sub-cellular level, the theory and practice of complex systems ecology has developed, for example, in relationship to the International Joint Commission of the Great Lakes (www.ijc.org) as well as adaptive environmental assessment and management (Tabor et al., 2002; Allen et al., 1991; Waltner-Toews et al., 2008). While not all the relevant outcomes considered are health-related in a conventional sense, they all relate to the broader definitions of health that grew out of the healthy communities and similar movements (Waltner-Toews and Wall, 1996; Waltner-Toews, 2000). Understanding the community function of single-celled organisms extends systems biology to molecular ecosystems biology (Raes and Bork, 2008). Addressing systems biology of persistent tuberculosis, Young et al. (2008) extended the systems biology boundaries to include host and pathogen populations with quantitative linkages across different scales between molecules, cells, tissues, and organisms (Fig. 2). Transmission dynamics of brucellosis in sheep and cattle (Zinsstag et al., 2005a) exclusively determine the burden of human brucellosis, while demonstrating how health of humans is related to agro-ecological systems (Fig. 3). Human brucellosis burden is directly linked to available feed resources, which determine livestock population dynamics. In this way the health of humans and animals is directly related to resource overuse and prevailing social and institutional systems. Similarly, biosecurity and biocontainment at the animal–human interface in industrial food animal production require ecosystemic considerations (Graham et al., 2008). The theory and practice of understanding and managing human activities in the context of social-ecological systems has been well-developed by members of The Resilience Alliance (www.resalliance.org)^1^ and was used extensively in the Millennium Ecosystem Assessment, including its work on human well-being outcomes (www.millenniumassessment.org).^2^ It is hence not difficult to relate health to social-ecological systems (SES) or so-called human–environment systems. These systems relate outcomes, which can also be outcomes of health and well-being, to systemic interactions which are primarily influenced by resources, governance and users in a given social, economic and political setting and related ecosystems (Ostrom, 2007) (Fig. 4). This has been further emphasized by Rock et al. (2009), who explicitly linked social science to animal–human connections. The National Institutes of Health (NIH) office of behavioural and social sciences research aims at bringing together in a systemic way behavioural-social-ecologic models and the molecular, cellular and ultimately physiological bases of health and disease to improve the public's health (Mabry et al., 2008). To improve public health, systems thinking requires understanding of societal structures and functions. This has been applied, for example, to tobacco control and pandemic planning by emphasizing the role of social contact networks (Leischow et al., 2008). Atun and Menabde (2009) demonstrated the importance of a detailed systemic analysis of health systems and the socio-political and cultural context, to understand policy resistance and hindered broad adoption and diffusion of the WHO recommended tuberculosis control strategy in Russia. Societal structure and function, through its health system performance, thus has a direct effect on health outcomes, e.g. the cure rate of tuberculosis. Further, ill-conceived tuberculosis control programmes and poor compliance with drug intake contribute to cause resistance to antimicrobials, which are mechanisms at the molecular level. In this way, the quality of a health system performance is connected across scales of systems biological categories, from populations, to individuals, to pathogens. In this way too, the linkage of health systems and systems biological categories provide insight into the understanding of the effectiveness of control efforts and the risk of multi drug resistant strains of *Mycobacterium tuberculosis* (Young et al., 2008) (Fig. 2). As one of the first countries, the Canadian government established in 2002 a programme for integrated surveillance of antibiotic resistance in humans and animals using a systemic approach (Canadian Integrated Programme for Antimicrobial Resistance Surveillance, CIPARS, www.phac-aspc.gc.ca/cipars-picra/index-eng.php) (Fig. 5).

**Fig. 6:**
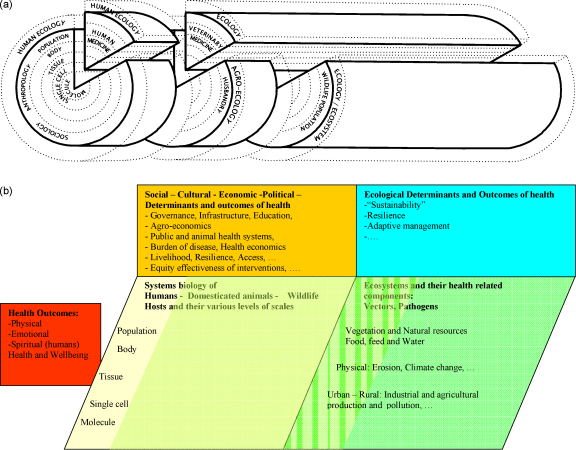
(a) Generalized systems dynamic framework of health of humans and animals in social-ecological systems extending the schematic used by Schwabe (1984). (b) Generalized systems dynamic framework of health of humans and animals extended from Ostrom (2007), Young et al. (2008) and Rock et al. (2009).

We extend here “one health”, “ecosystem health”, “systems biology” and SES conceptual thinking towards what we provisionally call “health in social-ecological systems” (HSES). In this way, we explicitly include health of humans and animals as a quantitative and qualitative interaction and outcome process in social-ecological systems (Ostrom, 2007) (Fig. 4). A graphical representation extends Schwabe's “one medicine” in Fig. 1, by structuring the different scales of systems biology (Fig. 2) as concentric circles and by adding the ecological and societal dimension as additional layers (Ostrom, 2007) (Fig. 6a). A similar graphical representation in Fig. 6b relates outcomes of health and well-being in humans and animals to systems biology of humans, domesticated animals and wildlife across scales from populations to molecules. As it relates to populations, it includes social, cultural, economic and political determinants of health. This interaction is mutual, as health determines social, cultural, economic and political outcomes of societies. Similarly, outcomes of health and well-being are related to ecosystems with their health-related components and vice-versa. Ecological determinants influence health and well-being of humans and animals, but at the same time ecosystem components are outcomes, also determined by the health of humans and animals. For example, livestock exports from Mongolia are banned because of endemic brucellosis, increasing the national herd, which in turn exerts heavy pressure on increasingly fragile pastures. In Fig. 6b, as an illustration, several concepts of social, development and ecological research are also listed. Animals and wildlife are part of the environment of humans, but are also part of the social systems of humans. Hence the distinction between social and ecological is flawed and is represented as a continuous green colour change. HSES is thus a comprehensive systemic approach to health of humans and animals. The system draws its boundaries at those physical, social and ecological issues that have no direct or indirect connection to human and animal health.

An example of a HSES approach is the issue of equity effectiveness of health interventions in humans and animals. Even if a vaccine or a drug has an excellent biological efficacy, its use in the field is often limited by a number of factors in a multiplicative way. As a result, effectiveness of an intervention, assessed as the proportion of humans and/or animal populations covered and cured or protected, may be largely below the actual biological curative or preventive efficacy of a drug or a vaccine. A vaccine's or drug's effectiveness (not its efficacy) in the field is determined by its availability, its accessibility and its affordability.

**Fig. 7:**
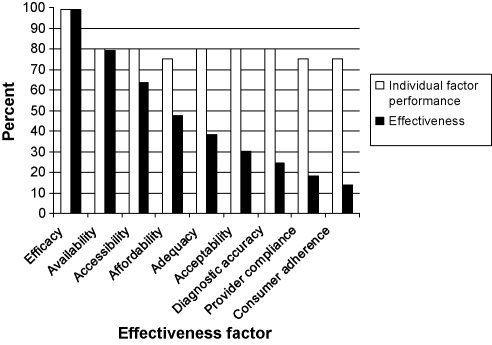
From efficacy to effectiveness, or how interventions lose traction (adapted from Zinsstag et al., 2011).

Control programmes need to be adequate and acceptable in different socio-cultural contexts, not forgetting diagnostic accuracy, health care provider compliance and consumer adherence (Fig. 7). Even if all influencing factors have a relatively high individual impact, their multiplicative effect means that interventions may, for example, drop below threshold coverage to interrupt transmission of a communicable disease. Interdisciplinary systemic research is required to assess the impact and variability of all individual factors. This will allow one to identify social (equity), gender, behavioural and health system dependent differentials (Tugwell et al., 2006). Identifying the most sensitive determinants of intervention effectiveness, combined with cross-sector economic analysis, provides insight into how to foster the effectiveness of an intervention to close the equity effectiveness loop (Tugwell et al., 2006). Such determinants can be a new drug, a new delivery system or a better understanding of a patient's behaviour, but also an institutional reform, increased health care capacity or a health financing reform. It can also mean reducing the availability of food to street dogs to control rabies, or developing household centred environmental sanitation to curb diarrhoea (Schertenleib, 2009). HSES will not only consider social and ecological determinants of health of humans and animals, but will also address social and ecological consequences of health interventions as part of an integrated development research approach. For example, brucellosis control in Kyrgyzstan and Mongolia involves not only issues of effective vaccine delivery, but also carrying capacity of pastures. This kind of research most often involves trans-disciplinary processes, which include communities, authorities, health practitioners and scientists, to identify sensitive determinants of health of humans and animals in a participatory and socially and culturally acceptable way (Schelling et al., 2007; Zinsstag, 2007).

## 8 Challenges ahead

On the one hand we struggle with persisting or even re-emerging infectious diseases. Rabies cases in Asia and in Africa increase despite well established knowledge of how to control this disease effectively. The persistence of anthrax in African livestock and humans because of poor quality of locally produced vaccines is in a stark contrast with the advancement in our understanding of the genomics of *Bacillus anthracis* motivated by bio-terrorism fears. For some diseases such as tuberculosis or brucellosis, despite all efforts we still have no better vaccines. Overall we observe a huge gap between knowledge and its application both in human and animal health delivery (Zinsstag, 2007).

On the other hand, time is short, as tremendous challenges, with unknown effects on health are looming on the horizon. But climate change and resource depletion become visible at an accelerated pace. Human hunger has reached unexpectedly high levels, influenced by the first commotions of an energy crisis, whose future dimensions are barely imaginable. How can we provide health care to still growing human and animal populations without losing all the gains due to menacing malnutrition, and how can we attempt to halt resource depletion? How do we deal with a devastating human resource crisis in human and animal health personnel? How do we provide health to a 2000 Watt society? How do we control trans-boundary diseases if surveillance systems are inadequate and barely operational? How do we control communicable diseases if available funds for control are diverted by corrupt authorities?

Solutions require all the possible intellectual imagination of science and technology, and at the same time new forms of cross-sectoral collaboration which involves all stakeholders. Insight into complex ecological and social processes will allow us to identify high leverage determinants of health and well-being of humans and animals. Issues of resource depletion and poor governance and their effects on health can only be addressed by international treaties and the development of civil societies. Enhanced North–South and South–South cooperation is now supported not only by development agencies but also by research funding agencies such as the Wellcome Trust or the Swiss National Science Foundation. Excellent experiences with international efforts such as the Pan African Rinderpest Campaign (PARC) or the Global Fund to fight HIV/AIDS, Tuberculosis and Malaria (GFATM) should be extended. Such successful past and ongoing initiatives provide inspiration and hope despite all odds. Nothing prevents global civil society to invent new, innovative public–private partnerships to pool resources for a global partnership to control trans-boundary animal diseases, zoonoses and neglected tropical diseases. Such endeavours are in the interest not only of developing countries but also of industrialized nations, as they will reduce the risk of introduction of such diseases worldwide.

## Conflict of interest statement

None declared.

## Footnotes

1 The Resilience Alliance was founded in 1999 by an international network of universities and government and non-government agencies, mainly in the fields of environmental sciences, ecology and economics.

2 The Millenium Ecosystem Assessment, convened by the United Nations Secretary-General Koffi Annan, assessed the consequences of ecosystem change for human well-being by over a thousand experts worldwide.
